# Real-Time Analysis of Antiproliferative Effects of Mouthwashes Containing Alcohol, Sodium Fluoride, Cetylpyridinium Chloride, and Chlorhexidine In Vitro

**DOI:** 10.1155/2021/2610122

**Published:** 2021-10-12

**Authors:** Mustafa Ülker, A. C. Tutku Çelik, Emine Yavuz, Firdevs Kahvecioğlu, H. Esra Ülker

**Affiliations:** ^1^Medicana Konya Hospital, Konya, Turkey; ^2^Private Celik Oral and Health Clinic, Istanbul, Turkey; ^3^Advanced Technology Research and Application Center, Selcuk University, Konya, Turkey; ^4^Department of Pediatric Dentistry, Faculty of Dentistry, Selcuk University, Konya, Turkey; ^5^Department of Restorative Dentistry, Faculty of Dentistry, Selcuk University, Konya, Turkey

## Abstract

**Objectives:**

In this study, the cytotoxic responses of six different over-the-counter mouthwashes on L929 cells were analyzed by two different techniques: the traditional colorimetric tetrazolium-based reduction assay (MTT) and the modern impedance-based real-time cell analysis (RTCA) system to investigate their biocompatibility in vitro. Thus, the investigation of the antiproliferative effects of the specified materials via different techniques is vital to reach this goal.

**Materials and Methods:**

First, L929 mouse fibroblasts were exposed to the dilutions of mouthwashes for 2 minutes. After incubation, the tetrazolium reduction method was used to assess the metabolic viability of cells measured by colorimetric MTT assay and morphological inspection of cells was performed via phase-contrast microscopy. Furthermore, the effect of each mouthwash on the proliferation, morphology, and adhesion of L929 cells was monitored continuously by a noninvasive and label-free RTCA system for 140 h.

**Results:**

Our data showed that all of the mouthwashes had varying cytotoxic effects on fibroblasts compared to the control group in MTT assay. In addition to that, RTCA technology has provided the growth kinetic profiles that can be used to analyze if the treatment is causing antimitotic or DNA-damaging effect on cells. Thus, analysis via this system can tell us the mechanism of toxicity behind the cell growth inhibition in vitro. Here, we found that only mouthwash 1 moderately maintained the viability of the L929 cells, yet displaying antimitotic effects and the other mouthwashes (mouthwash 2-mouthwash 6) showed toxicity via DNA-damaging effects.

**Conclusions:**

Of the six types of mouthwash tested, the most biocompatible result was obtained from a mouthwash containing alcohol (i.e., mouthwash 1). On the other hand, sodium fluoride- (NaF-) and cetylpyridinium chloride- (CPC-) containing mouthwash (i.e., mouthwash 2) showed the most cytotoxic effect.

## 1. Introduction

The dental plaque is a layer of bacterial complex and found naturally on the tooth surfaces which is the main cause of gingivitis, chronic periodontitis, and dental caries [1]. Daily tooth brushing with a fluoride-containing toothpaste and frequent usage of dental floss are the most commonly recommended methods for removing dental plaque [2]. However, besides the hard-to-reach places, the patients' efforts in oral hygiene remain at a critical level due to their inadequate skills and inadequate motivation. Thus, the use of antimicrobial mouthwashes with mechanical oral hygiene regimens is often considered a crucial combination to decrease dental plaque [3].

The quantity and diversity of chemical agents in mouthwashes are considerable, but many have antiseptic or antimicrobial effects, and their efficacy is highly variable [4]. Ideally, these types of substances should be biocompatible to tooth and oral tissues, reduce plaque formation and block the activity of microorganisms without altering the ratio between gram-positive and gram-negative anaerobic bacteria [5, 6]. The formulations based on antimicrobial agents provide noticeably greater preventive effect than the remedial effect on established plaque [4]. Since oral health contributes to total health, the maintenance of healthy oral soft and hard tissues is the most important aim of modern dentistry [7].

Commercial mouthwashes are produced for antiseptic, disinfectant, and protection purposes. In general, mouthwashes should have a combination of the following characteristics: effective against disease-creating habits, effective against disease-causing microorganisms where normal, healthy oral flora are not disrupted, safe for human and environmental use, minimal side effects, and likeable taste [6]. Chlorhexidine, essential oils, cetylpyridinium chloride, sodium fluoride, triclosan, octenidine, delmopinol, polyvinylpyrrolidone, hyaluronic acid, and other natural compounds are some of the frequently used supplements of common mouthwash formulations, and the complex composition of solutions often makes their cytotoxicity assessments inexplicit [6, 8].

Today, the best antiseptic for the oral cavity is chlorhexidine (CHX). CHX is a bacteriostatic agent at low concentrations, whereas it is bactericidal at higher concentrations [9]. CHX solution (0.2%) is often used as a standard, but researchers observed that a concentration of 0.12% was also clinically effective [10]. Cetylpyridinium chloride (CPC) is an amphiphilic quaternary compound with an antimicrobial activity facilitated by its positive charge that supports its binding to negatively charged bacterial surfaces which can also reduce bacterial adhesion on surfaces [11]. Essential oil-based mouthwashes usually contain a mixture of thymol, eucalyptol, menthol, methyl salicylate, and an alcohol vehicle that has antimicrobial and anti-inflammatory activities via antioxidant activity and considered the best alternative to CHX for plaque control. Also, one of the most commonly used mouthwash components is alcohol due to its capacity to preserve the formulation and dissolve the active ingredients. Moreover, NaF and SnF_2_ are the mainly used fluoride compounds in mouthwash formulations which affect oral bacteria due to their capacity to reduce bacterial acid metabolism [12, 13].

Mouthwashes interact with teeth and oral tissues during and after the application period. Given that, these products should not only be effective but also safe for oral tissue. Thus, biocompatibility studies are critically important for mouthwashes, like for other oral care products to prevent their destructive wrong use, especially now, when utilization of mouthwashes is strongly encouraged against human coronaviruses which may help to reduce the infection risk during recent coronavirus disease (COVID-19) pandemic [14].

In this study, the cytotoxic responses of six different over-the-counter mouthwashes were evaluated on L929 mouse fibroblast cells in vitro by two different quantitative techniques (i.e., widely used colorimetric formazan formation assay which is optimum for endpoint measurement of respiratory activity of the mitochondria and new technology xCELLigence RTCA for dynamic monitoring, respectively) and compared for toxicological risk assessment.

## 2. Materials and Methods

### 2.1. Cell Culture Condition and Mouthwash Dilutions

The six mouthwashes used in this study are as follows: M1 Listerine (Cool Citrus), M2 Colgate Plax, M3 Signal Expert Protection, M4 Oral B Proexpert, M5 Kloroben, and M6 Klorhex (Table 1). The dilutions of the mouthwashes were prepared under aseptic conditions. In each group, mouthwash was added to Dulbecco's Modified Eagle's Medium (DMEM, Biochrom GmBh, Berlin, Germany) and shaken gently to prepare the original stock solution (1 : 1). The stock solution was then serially diluted in DMEM until the dilution of 1 : 32 to provide different concentrations. L929 cells (ATCC CCL1, LGC Standards GmbH, Wesel, Germany), one of the standard cell lines frequently used in the cytotoxicity tests, were cultivated in DMEM supplemented with 10% Fetal Bovine Serum (FBS, Gibco Invitrogen, Karlsruhe, Germany) and 1% penicillin/streptomycin (Gibco Invitrogen, Karlsruhe, Germany) at 37 °C in a humidified atmosphere of 5% CO_2_.

### 2.2. MTT Assay

Percentage cell viability was determined by measuring the activity of mitochondrial enzymes such as succinate dehydrogenase by MTT (3-(4,5-dimethyl-thiazol-2-yl)-2,5-diphenyl-tetrazolium bromide) assay (Sigma-Aldrich, Steinheim, Germany). Briefly, L929 cells were seeded at a density of 10^4^ cells per well of a 96-well plate and incubated for 24 h at 37 °C. Afterward, the cells were exposed to either 200 *μ*L of mouthwashes of different dilutions or culture medium (i.e., negative control). After 2 min of incubation, L929 cells were washed with phosphate-buffered saline (PBS) twice to avoid any interference. Subsequently, 200 *μ*L aliquots of freshly prepared MTT solution (0.5 mg/mL in growth medium) were added to each well and incubated for 2 h at 37 °C. Then, the medium was removed, and blue formazan precipitate (reduced MTT) in each well was dissolved in 200 *μ*L of dimethyl sulfoxide (DMSO) on a shaker at room temperature for 20 min. The absorbance of samples against a blank (i.e., medium) at 540 nm (OD540) was measured spectrophotometrically by a multiwell plate reader (Epoch, BioTek, Winooski, VT, USA). Percentage cell viability was calculated as 100% × (*absorbance* of *treated* *cells* *against* *blank*)/(*absorbance* of *control* *against* *blank*). At the end of each treatment, images of cells were taken by an inverted microscope (Leica DM IL LED, Leica, Germany). Twelve wells were used for each group in two independent experiments (*n* = 24). The data were normally distributed. Results were statistically analyzed by one-way ANOVA followed by the Tukey-HSD test for post hoc comparisons (*α* = 0.05; SPSS version 13.0; SPSS, Chicago, IL).

### 2.3. RTCA Assay

Cell proliferation assays were performed using the xCELLigence real-time cell analyzer dual plate (RTCA DP, ACEA Biosciences, San Diego, CA) system. First, to perform a background measurement, 50 *μ*L of prewarmed DMEM was added to each well of the E-plate 16 (ACEA Biosciences, San Diego, CA, USA). L929 cells were then seeded at a density of 10^4^ into each well, and E-plates were placed in an RTCA DP analyzer. The cells adhered and proliferated in a 5% CO_2_ and 95% humidified incubator at 37 °C for approximately 21 h while the electrical impedance in the wells was measured every hour by a cell index (CI, an arbitrary unit). After that, the mouthwash extracts, which were prepared according to the LD50 (lethal dose 50) value of MTT assays, M1: 19%, M2: 12%, M3: 11%, M4: 54%, M5: 40%, and M6: 35%, were added following the predetermined layout in the software. CI measurements were taken every 15 min for 140 h. All treatments were performed at a volume of 150 *μ*L. Each group was studied with two replicates, and the results were analyzed by the RTCA 2.0 software.

## 3. Results

### 3.1. Morphology

**Figure 1:**
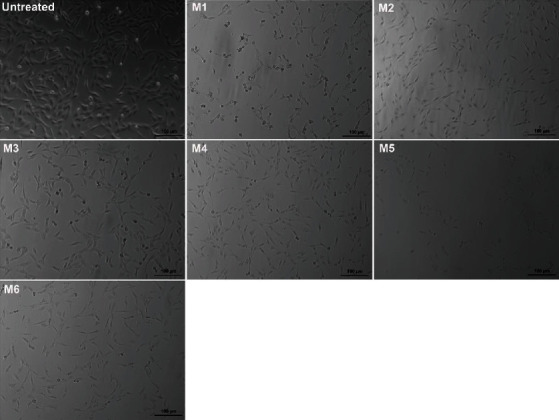
Representative microscopic images of L929 fibroblasts exposed to six different mouthwashes (M1-M6). Cultured L929 fibroblasts were exposed to a 1 : 1 dilution of mouthwashes for 2 minutes except for untreated control cells. Then, changes in cell morphology were observed via phase contrast microscopy. Bipolar round cell shape and cell detachment were observed after treatments. Images were taken at a 10x magnification. The scale bars represent 100 *μ*m.

As seen in Figure 1, overnight cultured untreated L929 cells showed regular spindle-shaped fibroblast morphology. However, a reduced cell density and inhibited cell growth were observed in the cells stimulated with mouthwashes (M1-M6) at 1 : 1 dilution in comparison with the control group. Small round-shaped cells and cell detachment were characteristics of treatment groups. Thus, mouthwashes can exhibit in vitro toxicity affecting their morphology and adherence.

### 3.2. Assessment of Cell Viability Using the MTT Assay

**Figure 2:**
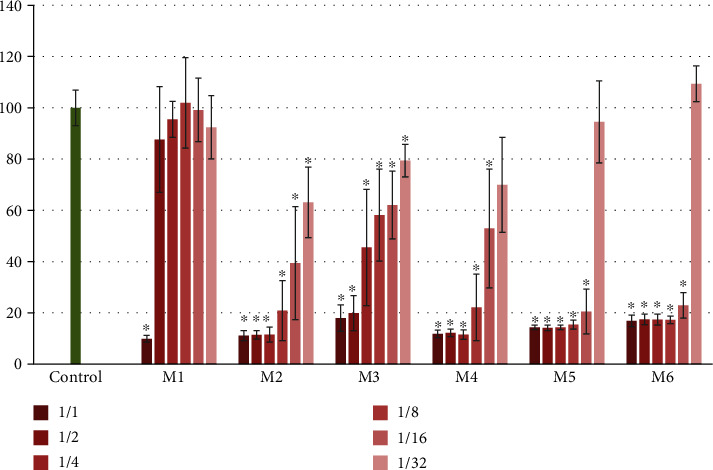
Relative cell viability of L929 cells treated with six different mouthwashes of different dilutions for 2 min. The control represents cells with no treatment. The values show percentages of cell viability (mean ± SD, *n* = 20). Significant difference with respect to control is denoted as ^∗^*p* < 0.05.

The mouthwashes used in this study were tested for toxicity toward L929 fibroblasts using MTT assay. The cell survival values of L929 fibroblasts for each group were expressed as percent cell viability where nontreated cells served as a control (set at 100% cell viability). As seen in Figure 2, a significant reduction in the cell viability was observed in all 1 : 1, 1 : 2, 1 : 4, 1 : 8, and 1 : 16 dilutions of M2, M3, M4, M5, and M6 (*p* < 0.05) which revealed a decrease in the mitochondrial functions of the cells following 2 min treatment. Moreover, the 1 : 1 dilution of all mouthwashes caused almost total inhibition (more than 80% reduction). Significantly, 1 : 1 dilution of M1; all dilutions of M2 and M3; 1 : 1, 1 : 2, 1 : 4, 1 : 8, and 1 : 16 dilutions of M4, M5, and M6 caused cytotoxicity compared to the control group (*p* < 0.05). Finally, the remaining dilutions of M1 and 1 : 32 dilution of M4, M5, and M6 displayed no toxicity.

### 3.3. Assessment of Cell Proliferation Rate Using the RTCA Assay

**Figure 3:**
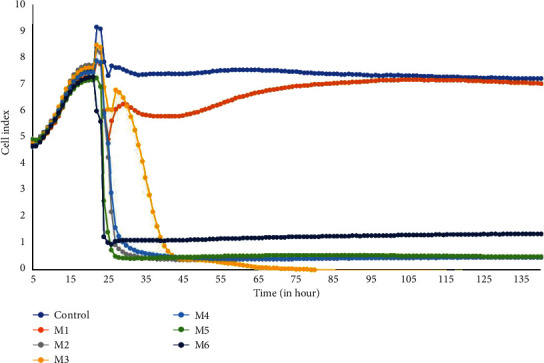
Dynamic monitoring of antiproliferative responses of L929 cells exposed to six different mouthwashes (M1-M6) using the RTCA system. The control shows cells without treatment. Different types of mouthwashes are indicated by different colors.

The potential antiproliferative effects of six different mouthwashes to L929 cells were studied via real-time monitoring of cell adherence, proliferation, and cell death using the xCELLigence RTCA system. The time-dependent effect of mouthwashes on L929 cells is shown in Figure 3. Significantly, the cells treated with M2, M4, M5, and M6 exhibited a rapid decrease in the CI value right after the addition of mouthwashes. Thus, M2, M3, M4, M5, and M6 showed DNA damaging effects on cells. On the other hand, M1 displayed a different kinetic response profile, as indicated by a moderate decrease in the CI compared to the control group over time which reflected its antimitotic effect. Although LD50 dose of mouthwashes after 24 h incubation in MTT assay was used in RTCA assay, the cell index values immediately dropped below 1 for all groups except M1 and M3. Overall, the tested mouthwashes revealed cytotoxic effects on L929 cells except for M1 as supported by the MTT assay results.

## 4. Discussion

This study was undertaken to evaluate the cytotoxic responses of the six different mouthwashes on L929 fibroblast cells using modern RTCA technology for the first time and refine the results with widely used traditional MTT assay. We believe that this manuscript is especially important in times of COVID-19 since some of the recent studies have demonstrated the virucidal properties of the over-the-counter mouthwashes to inactivate human coronaviruses [14, 15]. Despite their potential against the infectious virus, it is also urgent to inform the public about the possible adverse effects of oral hygiene products to prevent their detrimental misuse since these substances are in continuous contact with the periodontal tissue and alveolar bone and the substances released from mouthwashes may induce inflammation or necrosis.

To our knowledge, there have been very few published data in the literature using impedance-based label-free technologies on toxicity assessment of dental care products [16, 17]. RTCA analyzer is a unique tool that can continuously detect changes in cell number, viability, proliferation, and spreading of cells and provide dynamic intermediary cytotoxicity data that cannot be delivered in endpoint assays [18]. However, since the noninvasive RTCA measurements depend on the physical interaction of the cells with the gold microelectrodes in the wells, conventional assays such as MTT can be applied to provide some information regarding the toxicity mechanism (i.e., any impairment in the mitochondrial respiration activity of cells) [19]. Herein, the MTT assay demonstrated that all groups of mouthwashes, depending on dilutions, had different degrees of cytotoxic effects on L929 cells whereas M1 showed the most biocompatible results. Furthermore, M2, M3, M4, M5, and M6 revealed adverse cell proliferation effects in RTCA analysis which was consistent with the MTT assay results.

Importantly, we wanted to test if mouthwashes with similar compositions would have the same biocompatibility and which ingredients may cause a more potent toxic effect. Here, two of the tested mouthwashes (M1 and M3) contained sodium benzoate which is a preservative. According to MTT assay results, the 1 : 1 dilution of M1 inhibited the proliferation of L929 cells compared to the control group. On the other hand, all of the dilutions of M3 were cytotoxic for L929 cells. Both M1 and M3 contained sodium benzoate which may have caused cytotoxic effects since under acidic conditions, sodium benzoate is bacteriostatic and fungistatic [20]. Ishidate et al. also reported that sodium benzoate caused chromosome aberrations in the Chinese hamster fibroblast cell line [21]. In another in vitro study, the level of enzymes in the mitochondria and cytosol of rat liver hepatocytes was diminished by sodium benzoate when the concentration was higher than 500 *μ*g/mL and inhibited DNA synthesis at 100 *μ*g/mL [22]. However, Mpountoukas et al. studied the genotoxic, cytostatic, and cytotoxic potential of sodium benzoate in human peripheral blood cells in vitro and concluded that sodium benzoate did not induce any cytotoxicity and was nongenotoxic at low concentrations [23]. In addition, Zengin et al. studied the effects of sodium benzoate in cultured human peripheral lymphocytes. As compared with control, they have found that mitotic index values decreased, and chromosome aberrations increased in a dose- and time-dependent manner [24]. The results of our study also supported the importance of sodium benzoate concentration in cytotoxicity analysis since M1 showed little effect on the viability of L929 cells at lower concentrations in MTT assay and the cytotoxic effect of M3 may be due to another supplement (i.e., fluoride) found in its composition.

It was reported that high-alcohol mouthwashes may cause toxic effects on monolayer cultures of gingival fibroblasts [25]. However, Silverman and Wilder showed that alcohol-containing antimicrobial mouth rinses were safe, and there was no correlation between alcohol in mouth rinses and any adverse effects on oral tissues [26]. In addition, Moharamzadeh et al. tested the alcohol-based mouthwashes on the 3D human oral mucosal model and did not reveal any significant cytotoxic damage [27]. Supported by these studies, we also found that alcohol-containing M1 showed the most biocompatible characteristics within the mouthwashes tested in this study.

The dental caries is affected by the sensitivity of the tooth surface, oral bacterial profile, the quality and quantity of saliva, and the fluoride (natural mineral) which supports remineralization and inhibits demineralization of the tooth's enamel layer [3]. Inorganic and organic fluorides can be found in many dental products [2, 5]. The most commonly used fluoride derivative in mouthwashes is sodium fluoride (NaF) [28, 29].

PEG-40 (PEGylated hydrogenated castor oil) is used in mouthwashes, cosmetics, and beauty products as an emulsifier, surfactant, and fragrance as a safe ingredient. In contrast, Müller et al. reported that oral rinse Tebodont Mundspülung, which contained PEG and propylene glycol, showed high cytotoxicity but no antimicrobial activity [8]. In this study, M3 contained PEG-40 and all of its dilutions showed dramatic toxic effects on L929 cells, but this may not directly attributable to PEG-40 since M3 had fluoride as well.

Poloxamer 407 is a hydrophilic nonionic surfactant of copolymers known as poloxamers. In cosmetics, it is usually used for dissolving oily materials in water. Previous studies have indicated that poloxamer 407 by itself does not have any adverse effects on rat tissues [30]. In our study, M1, M2, and M4 contained poloxamer 407 which showed varying degrees of toxic effects at different dilutions.

Cetylpyridinium chloride (CPC) is a cationic surfactant with plaque and calculus inhibitory effect and a wide antimicrobial spectrum of rapid killing of gram-positive pathogens and yeasts [6]. Besides, studies are showing the destructive effect of CPC on viruses which is widely used in personal care products and also used for respiratory infections. CPC promotes virus inactivation by destroying viral capsid with its lysosomotropic effect, which is common for quaternary ammonium compounds [11]. In our study, M2 and M4 contained CPC. All dilutions of M2 and all dilutions except 1 : 32 dilution of M4 were cytotoxic on L929 cells. In general, the quaternary ammonium salts with a positively charged nitrogen atom make them favorable to be uptaken by mitochondria which are critical cellular organelles responsible for energy formation and cellular homeostasis of cells. Datta et al. showed that CPC had mitochondrial inhibitory activity in vitro via inhibition of mitochondrial O_2_ consumption and ATP synthesis [31], which could impair the mitochondrial activity detected by MTT assay. Importantly, our results are consistent with the studies mentioned above.

Chlorhexidine (CHX) is a bisbiguanide antiseptic against microorganisms and viruses. Chlorhexidine mouthwashes were effective in controlling gingivitis and reducing plaque formation and dental caries [6]. Eren et al. found higher DNA damage in buccal cells than blood cells after CHX exposure via alkaline comet assay [32]. Hidalgo and Dominguez proposed that CHX inhibited mitochondrial activity, protein and DNA synthesis, and cell proliferation via cell death by ATP depletion [33]. Faria et al. suggested that CHX-induced endoplasmic reticulum (ER) stress may one of the factors causing cell death in L929 cells [34]. In this study, all dilutions except 1 : 32 dilution of M5 and M6 were cytotoxic on L929 cells which contained CHX. Furthermore, studies showed that CHX reduced cell proliferation in a dose- and time-dependent manner, and a high concentration of CHX induced irreversible cell damage and immediate cell death [35, 36].

Benzydamine hydrochloride- (BH-) containing mouthwashes can be used for their nonsteroidal anti-inflammatory activity in treatments [37]. Erciyas et al. investigated the genotoxicity of CHX- and BH-containing mouthwashes and their effect on survival rate in *Drosophila melanogaster* larvae and found the results supporting their higher genotoxicity and lower survivability [38]. Although the CHX concentration of M6 was higher than M5 containing BH, our findings revealed a similar toxic effect in vitro. This may attribute to its BH content. Moreover, CHX-containing M5 and M6 showed more toxic values than alcohol-containing M1. Similarly, a comparative study showed that 0.2% CHX was more cytotoxic than alcohol-containing mouthwash on human gingival fibroblasts [39].

## 5. Conclusion

In conclusion, despite the evidence shown here that the mouthwashes can be cytotoxic in vitro, it should be noted that this may not be transferable to the buccal cavity due to the complexity of the oral environment with different variables such as host immunity, saliva, pH, and enzymes. Thus, further studies are necessary to assess the biocompatibility of mouthwashes in microenvironment of oral cavity. Here, we want to study whether potentially incompatible ingredients can be found in mouthwashes. However, it is also not easy to reach a clear conclusion since the exact compositions of mouthwashes are not publicly shared. Consequently, more conclusive in vitro and clinical data are still needed.

## Figures and Tables

**Table 1:** Compositions of different mouthwashes investigated for in vitro cytotoxicity.

Code	Material	COMPOSITION
M1	Listerine (Cool Citrus) Lot No. 587630	Aqua, alcohol, sorbitol, aroma, poloxamer 407, benzoic acid, eucalyptol, methyl salicylate, thymol, sucralose, menthol, sodium benzoate, glycerin, Cl 75470
M2	Colgate Plax Lot No. 2342TH1134	Aqua, sorbitol, aroma, menthol, glycerin, poloxamer 407, propylene glycol, cetylpyridinium chloride, sodium fluoride, methyl paraben, Cl 42051, sodium saccharin
M3	Signal Expert Protection Lot No. 5CYC	Aqua, sorbitol, aroma, glycine, PEG-40 hydrogenated castor oil, potassium citrate, sodium benzoate, citric acid, zinc sulfate, sodium saccharin, limonene, Cl 17200, sodium fluoride
M4	Oral B Proexpert Lot No. 2354028811	Aqua, aroma, glycerin, cetylpyridinium chloride, poloxamer 407, methylparaben, sodium saccharin, propylparaben, eugenol, Cl 42090
M5	Kloroben Lot No. 14B015411	Sorbitol, propylene glycol, ecocool MP, mint, patent V blue, quinoline yellow, sucralose, citric acid monohydrate, sodium citrate monohydrate, 0.15% benzydamine HCl, 0.12% chlorhexidine gluconate
M6	Klorhex Lot No. 16I070411	% 0.2 chlorhexidine gluconate

## Data Availability

The data used to support the findings of this study are included within the article.
